# Neuroendocrine neoplasms of the breast: diagnostic agreement and impact on outcome

**DOI:** 10.1007/s00428-022-03426-0

**Published:** 2022-10-15

**Authors:** Jasna Metovic, Eliano Cascardi, Silvia Uccella, Roberta Maragliano, Giulia Querzoli, Simona Osella-Abate, Alessandra Pittaro, Stefano La Rosa, Giuseppe Bogina, Paola Cassoni, Caterina Marchiò, Anna Sapino, Isabella Castellano, Mauro Papotti

**Affiliations:** 1 Department of Oncology, Pathology Unit, University of Turin, Turin, Italy; 2 Candiolo Cancer Institute, Pathology Division, FPO-IRCCS, Candiolo, Italy; 3 Department of Medicine and Surgery, Unit of Pathology, University of Insubria, Varese, Italy; 4 Department of Pathology, IRCCS Sacro Cuore Don Calabria Hospital, Negrar Di Valpolicella, Verona, Italy; 5 Department of Medical Sciences, Pathology Unit, Città Della Salute E Della Scienza Hospital, Turin, Italy; 6 Department of Medical Sciences, Pathology Unit, University of Turin, Turin, Italy

**Keywords:** Neuroendocrine, Breast cancer, NET, NEC, Diagnosis, WHO 2019

## Abstract

The classification of breast neuroendocrine neoplasms (Br-NENs) was modified many times over the years and is still a matter of discussion. In the present study, we aimed to evaluate the diagnostic reproducibility and impact on patient outcomes of the most recent WHO 2019 edition of breast tumor classification, namely, for neuroendocrine tumors (NETs) and neuroendocrine carcinomas (NECs). This multicentric observational study included 287 breast neoplasms with NE differentiation. The cases were blindly classified by three independent groups of dedicated breast and/or endocrine pathologists following the 2019 guidelines. Diagnostic concordance and clinical impact were assessed. We observed only a moderate overall diagnostic agreement across the three centers (Cohen’s kappa 0.4532) in distinguishing NET from solid papillary carcinomas (SPCs) and no special type carcinomas (NST) with NE differentiation. Br-NENs were diagnosed in 122/287 (42.5%) cases, subclassified as 11 NET G1 (3.8%), 84 NET G2 (29.3%), and 27 NEC (9.4%), the latter group consisting of 26 large-cell and 1 small-cell NECs. The remaining 165/287 (57.5%) cases were labeled as non-NEN, including SPC, mucinous, NST, and mixed NE carcinomas. While NET and non-NEN cases had a comparable outcome, the diagnosis of NECs showed negative impact on disease-free interval compared to NETs and non-NENs (*p* = 0.0109). In conclusion, the current diagnostic classification of Br-NENs needs further adjustments regarding morphological and immunohistochemical criteria to increase the diagnostic reproducibility among pathologists. Our data suggest that, apart from high-grade small- and large-cell NECs, Br-NENs behave like non-NEN breast carcinomas and should be managed similarly.

**Supplementary information:**

The online version contains supplementary material available at 10.1007/s00428-022-03426-0.

## Introduction

Over the years, the classification of neuroendocrine neoplasms of the breast (Br-NENs) has been updated by the multiple WHO editions and is still under discussion. These changing diagnostic criteria reflect the uncertainty about the real clinical impact of these rare tumors.

In 2018, Rindi et al. [1] and the latest WHO classification of endocrine and neuroendocrine tumors proposed a common classification framework for neuroendocrine neoplasms (NEN) found in any anatomical location [2]. The intent was to allow consistent patient management, while acknowledging organ-specific differences in classification criteria, tumor biology, and prognostic factors. Following this proposal, the updated NEN classification was also introduced in the 2019 WHO breast tumor fascicle [3], recommending pathologists adopt the terms “neuroendocrine tumor” (NET) and “neuroendocrine carcinoma” (NEC) in cases showing the morphology and immunoprofile typical of, respectively, well-differentiated and poorly differentiated NENs. According to the Elston and Ellis grading system, Br-NENs are graded as well-differentiated tumor (G1), intermediate differentiated tumor (G2), or poorly differentiated (G3) carcinoma. To completely adhere with this classification framework, special-type breast carcinomas (BCs) expressing neuroendocrine markers, such as solid papillary carcinomas (SPCs) and mucinous carcinomas, were removed from the NEN category [3].

Although a uniform classification of all NENs from different organ systems may represent an ideal approach for both pathologists and clinicians, its application to breast neoplasms has raised several uncertainties [4, 5]. Indeed, there is still a lack of clear-cut standards to differentiate real Br-NENs from solid BCs having some degree of neuroendocrine differentiation. An effective differential diagnosis requires validated and reproducible morphological criteria, in addition to well-defined qualitative and quantitative thresholds for neuroendocrine marker assessment [4]. Regarding prognosis, only few studies described the impact of NE differentiation in BC, reporting contrasting results [6, 7]. From a clinical perspective, a diagnosis of Br-NET, despite the term *tumor*, implies an identical treatment to any BC of comparable grade, stage, and hormonal profile, being most Br-NETs estrogen (ER) and progesterone (PgR) receptor positive.

In this complex background, the aim of our study was twofold: first, we evaluated the diagnostic agreement for Br-NENs among dedicated breast and endocrine pathologists strictly adhering to the latest WHO edition, reviewing a series of BC diagnosed as “neuroendocrine” or “with neuroendocrine features” from 2001 to 2019. Second, we analyzed the impact of the diagnosis of Br-NET and Br-NEC on patient outcomes to better understand the clinical relevance of these peculiar entities.

## Materials and methods

### Case series

To select a series of BC with NE differentiation, we searched the electronic medical records for keywords such as “breast,” “carcinoma,” “neoplasm,” “infiltrative,” and “neuroendocrine” and included surgical specimens with available hematoxylin and eosin (H&E)–stained glass slide and at least one general neuroendocrine marker immunostain. Specifically, we collected and included 100 specimens of BC with NE differentiation from Città della Salute e della Scienza Hospital (Turin, Italy), 23 from Candiolo Cancer Institute (Candiolo, Italy), 75 from University of Insubria (Varese, Italy), and 89 from Sacro Cuore Don Calabria Hospital (Verona, Italy), totaling 287 cases.

For each case, available demographic and clinical data such as age, disease stage, type of surgery, type of therapy, and follow-up data were obtained from clinical charts. Pathological report information included tumor diameter, histological grade, mitotic count, surgical margins status, vascular invasion, Ki67 proliferation index, as well as ER, PgR, and human epidermal growth factor receptor 2 (HER2) status.

Both ER and PgR were considered positive if more than 1% of tumor cells had a nuclear immunostaining [8]. HER2 status was classified as negative (score 0, 1 + , or 2 + not amplified) or positive (score 3 + by IHC or HER2 amplified by FISH) according to the recommended guidelines for invasive carcinoma [9].

The data collection also included surrogate molecular profiles based on immunohistochemical/FISH status of ER, PgR, HER2, and Ki67 according to the recommendations of St. Gallen [10]. Immunohistochemical data regarding the percentage of positivity of chromogranin A (CgA) and synaptophysin (SYN) were also recorded.

The study was approved by the Research Ethics Committee for Human Biospecimen Utilization (Department of Medical Sciences—ChBU) of the University of Turin (n°5/2020) and by The Ethics Committee of Candiolo Cancer Institute (“Neurobreast” project). Written consent was not required considering the retrospective nature of the study and no impact on patients’ care. The study was conducted in accordance with The Code of Ethics of the World Medical Association (Declaration of Helsinki). All cases were de-identified, and all clinical-pathological data were accessed anonymously.

### Case review

For each of the 287 cases, a representative H&E-stained section and at least one neuroendocrine marker (CgA and/or SYN) were included in the blind review process (of the original diagnosis) by three independent groups of experts from Turin, Candiolo, and Varese (henceforth regarded as A, B, and C research centers, respectively). The groups were composed of breast and/or endocrine dedicated pathologists, instructed to strictly follow the criteria stated in the 2019 WHO fifth edition of breast tumor classification [3].

The presence of convincing neuroendocrine morphology in > 90% of the tumor area was required for the diagnosis of NET or NEC. The diagnosis of NET was rendered in cases of invasive tumor with low- to intermediate-grade neuroendocrine morphology, showing organoid growth patterns (solid nests, trabeculae, pseudoglands) and typical cytology, while NEC cases were bearing high-grade neuroendocrine morphology, almost indistinguishable from their pulmonary small-/large-cell counterpart. Diffuse and uniform immunoreactivity for neuroendocrine markers was required in support of diagnosis, both for NET and for NEC.

Cases with general NE marker expression, in which clear and indicative NE morphology according to the latest WHO criteria was not observed, were defined as non-NENs and included no special type (NST) carcinomas and special types of BC, including SPC and mucinous carcinoma. Cases showing co-existence of NEN and non-NEN where both components comprised 10 to 90% of the tumor area, were designated as mixed carcinomas. Before the revision process started, we agreed that a diagnosis was acceptable when at least two reviewer groups selected the same diagnostic category. Cases with complete discordance between three groups were reviewed by a fourth, independent reviewer, followed by a consensus meeting among all participants using digital images, to reach agreement.

### Statistical analysis

Statistical analyses were carried out using Stata 15.0 software (StataCorp, College Station, TX, USA).

To evaluate the consensus between rendered diagnoses, the “overall agreement” was calculated, i.e., the sum of the true positives and true negatives was compared to the total number of cases by calculating Cohen’s kappa index to eliminate the random component. The interpretation of the kappa values was performed according to the following guidelines: *k* 0.01–0.20 = none to slight agreement; *k* 0.21–0.40 = fair agreement; *k* 0.41–0.60 = moderate; *k* 0.61–0.80 = substantial; *k* 0.81–1.00 = almost perfect agreement [11].

The predominant NEN histotype diagnosis was used to study the clinical impact. The differences in the clinical and pathological variables were analyzed using parametric and non-parametric tests (Student’s *t* test, Pearson’s chi-square test, Bonferroni’s correction, Wilcoxon’s rank test).

Disease-free survival (DFS) was assessed from the date of diagnosis to the date of relapse or the date of the last checkup. Overall survival (OS) was assessed from the date of diagnosis to the date of death from any cause or to the date of the last checkup. All deceased patients were considered events. The survival analysis was determined by the Kaplan–Meier curves, and the Mantel log-rank test was used to compare the statistical differences. Univariable and multivariable Cox regression analyses were carried out on DFS and OS to calculate HRs and 95% CIs for the different study groups. The proportional hazard assumption was assessed with the Schoenfeld residuals. This did not give reasons to suspect violation of this assumption. All statistical tests were two sided. *P* values < 0.05 were considered significant.

## Results

**Fig. 1:**
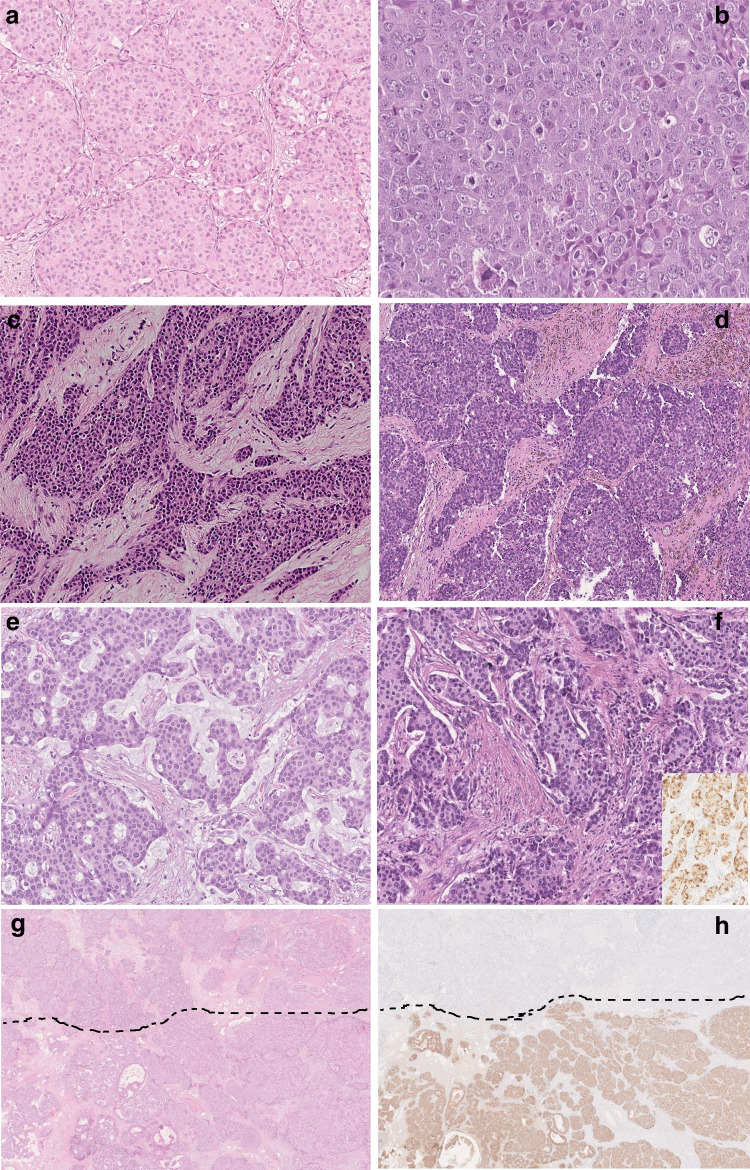
A case of NET G2, demonstrating organoid growth pattern, with solid nests separated by a thin fibrovascular stroma (**a**, 100 ×). High-grade NECs (**b**, **c**, 200 ×) showing large/pleomorphic (**b**) and small/lymphocyte-like cells (**c**) with hyperchromatic nuclei, and high mitotic index. Solid papillary carcinoma (**d**, 100 ×) with typical solid-growth pattern, delicate fibro-vascular cores, and mild nuclear atypia. Sheets of neoplastic cells suspended in abundant extracellular mucin, typically seen in mucinous breast carcinoma (**e**, 100 ×). Invasive carcinoma of no special type (**f**, 100 ×) showing neuroendocrine differentiation as revealed by chromogranin A immunohistochemical analysis (insert, 150 ×). A case of mixed breast carcinoma neatly divided into two components, upper NST and lower NET G2 (**g**, 10 ×), showing absence and strong expression of chromogranin A, respectively (**h**, 10 ×)

According to the latest WHO classification, Br-NENs were diagnosed in 122/287 (42.5%) cases. These were subclassified as NET G1 (11 cases, 3.8%), NET G2 (84, 29.3%), and NEC (27, 9.4%). The NEC group consisted of 26 large-cell and 1 small-cell carcinomas. The remaining 165/287 (57.5%) cases included tumors that did not meet the strict morphological NEN criteria such as NST (58 cases, 20.2%), SPC (30 cases, 10.5%), mucinous carcinomas (50 cases, 17.4%), and mixed-type BCs (27 cases, 9.4%) (Table 1, Fig. 1).

**Table 1:** Diagnostic classification of a series of 287 breast carcinomas with neuroendocrine differentiation

	NET	NEC	Other histotypes
NET G1	11		
NET G2	84		
NEC G3 (LCNEC)		26	
NEC G3 (SCNEC)		1	
Mixed carcinoma			27
Solid papillary carcinoma			30
Mucinous carcinoma			50
NST carcinoma			58
Total	95	27	165

### Multicentric case revision: inter-reviewers’ agreement

The three centers (A, B, and C) reached complete agreement in 122/287 (42.5%) cases, partial agreement in 126/287 (43.9%) cases (where two of three centers rendered the same diagnosis), and completely discordant diagnoses in 39/287 (13.6%) cases, most of them showing solid growth and/or mucinous foci. The diagnostic discrepancies among centers are shown in detail in Supplementary Table 1.

After the consensus discussion among all reviewers using digitalized glass slides, the discrepant cases were independently reviewed by a fourth pathologist (AP), using a relative diagnostic majority to evaluate the clinical relevance of the Br-NEN histotype (see “Materials and methods” section).

We observed a moderate diagnostic agreement across all three centers (Cohen’s kappa 0.4532) (Table 2). Comparing each center, we noted substantial agreement between centers A and B (Cohen’s kappa 0.6329, 70.4%), while a fair agreement was observed between centers A and C (Cohen’s kappa 0.4004, 50.87%), and centers B and C (Cohen’s kappa 0.3442, 46.7%) (Table 2).

**Table 2:** The inter-center case revision reproducibility

Research center	Agreement (%)	Expected agreement (%)	Cohen’s kappa	Description	Cohen’s kappa combined across 3 centers
A/B	70.4	19.3	0.6329	Substantial agreement	0.4532
A/C	50.87	18.1	0.4004	Fair agreement	
B/C	46.7	18.7	0.3442	Fair agreement	

### Clinico-pathological characteristics of the case series

Table 3 shows the clinico-pathological characteristics of the case series. We observed a significantly larger median diameter in NECs (19 mm), compared to NET (12 mm) and non-NENs (15 mm) (*p* = 0.042). Likewise, a significantly higher median mitotic count in NECs (13 mitoses/mm^2^), compared to NETs (4 mitoses/mm^2^) and non-NENs (5 mitoses/mm^2^) (*p* < 0.001).

**Table 3:** Clinico-pathological characteristics of the case series, according to the WHO 2019 diagnostic classification

		Total	NET (X)	NEC (Y)	NON-NEN (Z)	*p* value
Age	Median (interval)	72 (35–93)	73 (43–91)	66 (38–92)	72 (35–89)	0.592
Tumor diameter (275 cases)	Median mm (interval)	14 (0.3–140)	12 (0.3–120)	19 (1.9–65)	15 (0.5–140)	0.042
Number of mitoses (mm^2^)	Median (interval)	5 (0–90)	4 (1–20)	13 (7–42)	5 (0–90)	< 0.001
Stage (198 cases)	1	102	37	6	59	0.231
	2	72	18	8	46	
	3	17	4	3	10	
	4	7	2	2	3	
Grade (281 cases)	1	43	11	0	32	< 0.0001 (Y vs. X < 0.0001; Z vs. Y < 0.0001)
	2	191	84	0	107	
	3	47	0	27	20	
Surgical margins (280 cases)	Negative	249	87	18	144	0.006 (Y vs. X 0.004; Z vs. Y 0.044)
	Positive	31	5	7	19	
Vascular invasion (275 cases)	Absent	169	53	12	104	0.187
	Present	106	38	13	55	
HER2 status (272 cases)	0	172	56	18	98	0.455
	1	62	25	2	35	
	2	31	7	4	20	
	3	7	2	1	4	
HER2 FISH	Not amplified	21	2	4	15	0.395
	Amplified	5	0	0	5	
Chromogranin A	Median % (interval)^a^	0 (0–100)	1 (0–100)	5 (0–98)	0 (0–100)	0.345
Chromogranin A (215 cases)	Negative	89	26	7	56	0.421
	positive	126	45	13	68	
Synaptophysin	Median % (interval)^a^	80 (0–100)	90 (0–100)	90 (50–100)	70 (0–100)	0.003
Synaptophysin (253 cases)	Negative	4	2	0	2	0.697
	Positive	249	84	24	141	
Radiotherapy (252 cases)	No	131	36	17	78	0.142
	Yes	121	45	9	67	
Chemotherapy (249 cases)	No	126	46	9	71	0.120
	Yes	123	34	17	72	
Estrogen receptor	Median % (interval)^a^	98 (0–100)	99 (8–100)	96.5 (0–100)	95 (0–100)	0.033
Progesterone receptor	Median % (interval)^a^	75 (0–100)	81 (0–100)	50 (0–100)	70 (0–100)	0.006
Ki67	Median % (interval)^a^	17 (1–80)	14 (1–62)	31 (10–80)	16 (2–80)	< 0.001
Molecular surrogate classification	Luminal A^b^	113	41	5	67	0.008
	Luminal B^b^	85	20	17	48	
	Others	10	2	1	7	
Recurrent disease (231 cases)	No	198	65	15	118	0.043 (Y vs. X 0.043)
	Yes	33	8	7	18	
Follow-up status (257 cases)	Alive	200	65	19	116	0.828
	Dead	57	18	7	32	

^a^Median value refers to % of estrogen receptor, progesterone receptor, Ki67, chromogranin A, and synaptophysin-positive cells

^b^Molecular surrogate classification was based on St. Gallen recommendations [10]. Luminal A: ER and PgR positive/HER2 negative/Ki-67 low. Luminal B (HER2 negative): ER positive/HER2 negative and at least one of Ki-67 high and/or PgR negative or low. Luminal B-like (HER2 positive): ER positive/HER2 over-expressed or amplified/any Ki-67/any PgR

Almost one-third of NECs’ surgical margins were positive (28%, 7/25), while the surgical margin involvement was seldomly seen in NETs (5.7%, 5/87) and non-NENs (13.2%, 19/144) (*p* = 0.006).

The median value of SYN expression was significantly higher in NECs (90, interval 50–100), compared to NETs (90, interval 0–100) and non-NENs (70, interval 0–100) (*p* = 0.003), being positive in nearly all NEC cases with available staining. We did not observe significant differences in the expression of CgA across the groups (Table 3).

Regarding surrogate molecular classification, virtually all Br-NENs in our series belonged to luminal subtypes. In detail, luminal A subtype was more frequent in NETs (41/63, 65.1%), compared to NECs (5/23, 21.7%) and non-NENs (67/122, 54.9%). In contrast, luminal B subtype was more frequent in NECs (17/23 cases, 73.9%) than NETs (20/63, 31.7%) and non-NENs (48/122, 39.2%) (*p* = 0.008). All BCs, regardless of the presence or absence of neuroendocrine differentiation, were treated according to the surrogate molecular classification.

In comparison to NECs, which show recurrent disease in one-third of affected patients (7/22, 31.8%), recurrence was less common in NETs (8/73, 11%) and non-NENs (18/126, 14.3%) (*p* = 0.043) (Table 3).

### Impact of WHO 2019 Br-NEN classification on the outcome

**Fig. 2:**
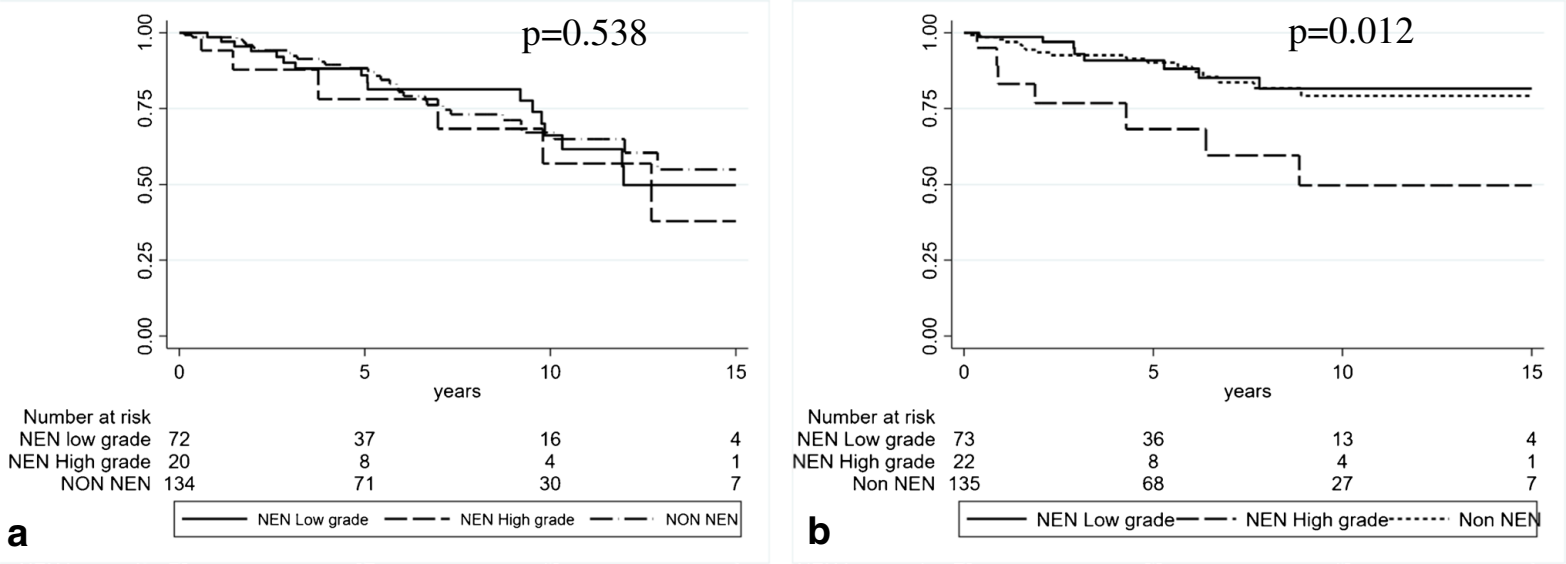
Kaplan–Meier estimates of overall survival (*p* = 0.538) (**a**) and disease-free survival (*p* = 0.012) (**b**) and according to breast NEN and non-NEN diagnostic categories

In univariate analyses and Kaplan–Meier estimates, no differences were observed in OS. However, when DFS was considered, the diagnosis of NEC (HR 3.67, CI 1.33–10.1, *p* = 0.012) was associated with an adverse prognosis (Supplementary Table 2; Fig. 2a, b).

In detail, as shown in Supplementary Table 3, there is a significant declining trend in 5- and 10-year DFS. At 5-year assessment, 91.1% of NETs and 90.2% of non-NENs avoided recurrent disease, compared to 69.6% of NECs. Furthermore, 81.9% of patients with NETs and 79.5% of non-NENs were disease free at 10-year follow-up, versus 50.3% of patients with NECs (*p* = 0.0109) (Supplementary Table 3).

However, DFS multivariate analyses that included tumor diameter, grade, and classification according to the WHO 2019 did not show a significant impact of the latter on the outcome (Supplementary Table 4).

## Discussion

Intending to increase diagnostic reproducibility, WHO’s latest edition of breast tumors classification [3] employs a uniform scheme for all human NE tumors across the different organ systems, as proposed by the International Agency for Research on Cancer [1]. However, when applied to the diagnostic practice, this classification has proven to be difficult to manage, and the existence of a pure Br-NEN is still controversial and widely debated in the literature, except for the extremely rare primary small cell carcinoma [12–14]. Indeed, in our experience, we can apply the category of pure Br-NENs in a small subset of cases, whereas mixed forms, in which NEN component co-exists with a NST or special type BC, are more common. However, although mixed neoplasms (mixed neuroendocrine/non-neuroendocrine neoplasms, MiNEN) are considered an integral part of NENs in the digestive tract [15] and other organs [1, 2], the WHO classification of breast tumors does not include this entity as a NEN [3].

As recently discussed in several studies [4, 13, 14], fitting Br-NENs into the rigid two-tiered scheme of NEN classification (well-differentiated NETs vs. poorly differentiated NECs) poses various relevant diagnostic and conceptual challenges. In fact, the attempt to align the classification criteria of Br-NEN with those of other organs has been questioned, mainly due to the overlapping clinical behavior and treatment approach with that of non-NE conventional breast cancers.

Moreover, although Br-NENs are currently described as neoplasms with a morphology and immunophenotype (CgA SYN, INSM1, etc.) similar to that of gastroenteropancreatic and pulmonary NENs, the nuclear features typically seen in extra-mammary organs (“salt and pepper” stippled chromatin and small nucleoli) are not frequently noted in Br-NENs [4, 16].

Considering the difficulties in rendering a diagnosis of Br-NEN, to the best of our knowledge, we performed the first multicentric study on the diagnostic reproducibility of a relatively large series of breast neoplasms with NE differentiation. We observed a moderate diagnostic agreement across all three centers (Cohen’s kappa 0.4532), although in approximately 13% of cases, a uniform diagnostic interpretation among the three research centers was not obtained. This suggests that the diagnostic criteria for recognizing Br-NENs, including NET and NEC histologic types, may not be unanimously interpreted. Our study also suggests that a different professional background may influence the subjective interpretation of these tumors, differing between dedicated breast pathologists and those more specifically involved in endocrine tumor diagnostics. Furthermore, following the last WHO edition criteria [3], less than half of cases were diagnosed as NENs (122/287, 42.5%), confirming the rarity of these breast neoplasms, at least in their “pure” forms.

Immunohistochemical analysis of NE biomarkers represent the gold standard in the diagnosis of these tumors [5, 17]. Together with the most sensitive and specific markers, CgA and SYN [18], a novel biomarker, INSM1, has been proposed as an accurate indicator of NE differentiation of BC to support NEN diagnosis [5, 19, 20]. However, these immunomarkers are not routinely assessed, reserved for when pathologists identify or suspect a NE morphology in H&E-stained slides. Moreover, none of these markers proved useful in distinguishing pure Br-NENs from other mammary carcinomas with NE differentiation [3]. As a result, the diagnostic criteria, as well as proposed cut-off for NE immunomarkers, and terminology of Br-NEN still vary in recent studies [6], making data comparison impractical.

From a prognostic point of view, high-grade NEC cases demonstrated a shorter DFS, compared to NETs and non-NENs (*p* = 0.0109). However, these data were not confirmed in multivariate analyses. Moreover, all cases of NECs were G3 neoplasms, demonstrating larger tumor diameter, higher mitotic and Ki67 index, more frequently positive surgical margins, and lower PgR levels than NETs and non-NENs, all features associated with an aggressive disease. The DFS between NETs and non-NENs were similar, and there were no significant OS differences detected across all histological groups. These data suggest that NENs behave similar to other invasive BCs and clinically should be managed equivalently. Our results should be interpreted considering some limitations: (1) the retrospective nature of the study design, offset by a large multicentric case series; (2) the exclusion of mixed carcinomas from Br-NEN category, due to the lack of clear criteria to diagnose this entity in the breast, in contrast to the MiNEN category used in other organs.

In conclusion, the classification of Br-NENs went through multiple modifications over the years and is still a matter of debate. The current WHO (3) diagnostic proposal for Br-NEN needs further adjustments to facilitate accurate recognition of these neoplasms and increase the diagnostic reproducibility among pathologists. To date, it is important to emphasize that Br-NENs, in regard to clinical management, should be treated as conventional BCs.

In line with this, extreme caution should be adopted when making treatment decisions at a multidisciplinary level. In fact, as remarked by WHO 2019 (3), the term NET should be interpreted as cancer and not simply as tumor, implying the same clinical, therapeutic, and prognostic characteristics of NST carcinomas of identical grade and stage, to avoid unjustified treatment de-escalation, with a potential impact on patients’ health.

## Supplementary information

Below is the link to the electronic supplementary material.

Supplementary file1 (DOCX 19 KB)

Supplementary file2 (DOCX 14 KB)

Supplementary file3 (DOCX 13 KB)

Supplementary file4 (DOCX 13 KB)

## Data Availability

The datasets generated and/or analyzed during the current study are available from the corresponding author on reasonable request.
