# Supplementary material for: Neuroendocrine neoplasms of the breast: diagnostic agreement and impact on outcome
Source: Virchows Arch. 2022 Oct 15;481(6):839–46. doi: 10.1007/s00428-022-03426-0 (PMC9734208; doi:10.1007/s00428-022-03426-0)
Supplement: Supplementary file 1 — Supplementary file1 (DOCX 19 KB) [file 428_2022_3426_MOESM1_ESM.docx]

Supplementary Table 1. Inter-center case revision reproducibility.

| **a)** |  | B | | | | | | | | |
| --- | --- | --- | --- | --- | --- | --- | --- | --- | --- | --- |
|  |  | NET G1 | NET G2 | NEC G3 (LCNEC) | NEC G3 (SCNEC) | MIXED | SPC | MUCINOUS | NST | TOTAL |
| A | NET G1 | 8 | 0 | 0 | 0 | 0 | 1 | 0 | 2 | 11 |
|  | NET G2 | 0 | 66 | 7 | 0 | 2 | 6 | 0 | 6 | 87 |
|  | NEC G3 (LCNEC) | 0 | 6 | 11 | 0 | 1 | 0 | 0 | 1 | 19 |
|  | NEC G3 (SCNEC) | 0 | 1 | 0 | 0 | 1 | 0 | 0 | 0 | 2 |
|  | MIXED | 1 | 8 | 3 | 0 | 14 | 2 | 1 | 4 | 33 |
|  | SPC | 0 | 5 | 0 | 0 | 2 | 25 | 1 | 3 | 36 |
|  | MUCINOUS | 0 | 0 | 0 | 0 | 2 | 2 | 43 | 0 | 47 |
|  | NST | 0 | 11 | 2 | 0 | 2 | 0 | 2 | 35 | 52 |
|  | TOTAL | 9 | 97 | 23 | 0 | 24 | 36 | 47 | 51 | 287 |

| **b)** |  | C | | | | | | | | |
| --- | --- | --- | --- | --- | --- | --- | --- | --- | --- | --- |
|  |  | NET G1 | NET G2 | NEC G3 (LCNEC) | NEC G3 (SCNEC) | MIXED | SPC | MUCINOUS | NST | TOTAL |
| A | NET G1 | 3 | 0 | 0 | 0 | 0 | 1 | 0 | 7 | 11 |
|  | NET G2 | 1 | 37 | 15 | 1 | 1 | 2 | 1 | 29 | 87 |
|  | NEC G3 (LCNEC) | 0 | 2 | 7 | 1 | 0 | 1 | 1 | 7 | 19 |
|  | NEC G3 (SCNEC) | 0 | 0 | 0 | 1 | 0 | 0 | 0 | 1 | 2 |
|  | MIXED | 0 | 8 | 4 | 0 | 2 | 0 | 7 | 12 | 33 |
|  | SPC | 0 | 8 | 3 | 0 | 1 | 12 | 2 | 10 | 36 |
|  | MUCINOUS | 0 | 1 | 0 | 0 | 0 | 0 | 43 | 3 | 47 |
|  | NST | 0 | 2 | 4 | 0 | 3 | 0 | 2 | 41 | 52 |
|  | TOTAL | 4 | 58 | 33 | 3 | 7 | 16 | 56 | 110 | 287 |

| **c)** |  | C | | | | | | | | |
| --- | --- | --- | --- | --- | --- | --- | --- | --- | --- | --- |
|  |  | NET G1 | NET G2 | NEC G3 (LCNEC) | NEC G3 (SCNEC) | MIXED | SPC | MUCINOUS | NST | TOTAL |
| B | NET G1 | 3 | 0 | 0 | 0 | 0 | 0 | 0 | 6 | 9 |
|  | NET G2 | 0 | 34 | 18 | 1 | 1 | 5 | 0 | 38 | 97 |
|  | NEC G3 (LCNEC) | 0 | 4 | 7 | 0 | 0 | 0 | 1 | 10 | 23 |
|  | NEC G3 (SCNEC) | 0 | 0 | 0 | 0 | 0 | 0 | 0 | 0 | 0 |
|  | MIXED | 0 | 2 | 2 | 3 | 3 | 1 | 10 | 6 | 24 |
|  | SPC | 1 | 11 | 2 | 1 | 1 | 9 | 3 | 9 | 36 |
|  | MUCINOUS | 0 | 2 | 0 | 0 | 0 | 0 | 41 | 4 | 47 |
|  | NST | 0 | 5 | 4 | 2 | 2 | 1 | 1 | 37 | 51 |
|  | TOTAL | 4 | 58 | 33 | 3 | 7 | 16 | 56 | 110 | 287 |
