# Supplementary material for: Neuroendocrine neoplasms of the breast: diagnostic agreement and impact on outcome
Source: Virchows Arch. 2022 Oct 15;481(6):839–46. doi: 10.1007/s00428-022-03426-0 (PMC9734208; doi:10.1007/s00428-022-03426-0)
Supplement: Supplementary file 2 — Supplementary file2 (DOCX 14 KB) [file 428_2022_3426_MOESM2_ESM.docx]

Supplementary Table 2. Univariate analyses.

|  | Parameters | Disease free survival | | | Overall survival | | |
| --- | --- | --- | --- | --- | --- | --- | --- |
|  |  | HR | CI | P value | HR | CI | P value |
| Classification | NET | 1 |  |  | 1 |  |  |
|  | NEC | 3.67 | 1.33-10.1 | 0.012 | 1.43 | 0.59-3.47 | 0.426 |
|  | NON-NEN | 1.15 | 0.49-2.67 | 0.738 | 0.99 | 0.50-1.61 | 0.720 |

HR: Hazard Ratio; CI: Confidence Interval
