# Supplementary material for: Neuroendocrine neoplasms of the breast: diagnostic agreement and impact on outcome
Source: Virchows Arch. 2022 Oct 15;481(6):839–46. doi: 10.1007/s00428-022-03426-0 (PMC9734208; doi:10.1007/s00428-022-03426-0)
Supplement: Supplementary file 3 — Supplementary file3 (DOCX 13 KB) [file 428_2022_3426_MOESM3_ESM.docx]

| DFS | NET | NEC | NON-NEN | P value |
| --- | --- | --- | --- | --- |
| 1 year | 98.6 | 84.6 | 97.7 | 0.0109 |
| 5 years | 91.1 | 69.6 | 90.2 |  |
| 10 years | 81.9 | 50.3 | 79.5 |  |

Supplementary Table 3. Disease free survival at 1-, 5- and 10-years according to the new WHO 2019 classification system.
