# Supplementary material for: Neuroendocrine neoplasms of the breast: diagnostic agreement and impact on outcome
Source: Virchows Arch. 2022 Oct 15;481(6):839–46. doi: 10.1007/s00428-022-03426-0 (PMC9734208; doi:10.1007/s00428-022-03426-0)
Supplement: Supplementary file 4 — Supplementary file4 (DOCX 13 KB) [file 428_2022_3426_MOESM4_ESM.docx]

Supplementary Table 4. Multivariate analysis.

| Disease free survival |  | HR | CI | P value |
| --- | --- | --- | --- | --- |
| Tumor diameter |  | 1.02 | 1.01-1.03 | 0.004 |
| Grade | 1 | 1 |  |  |
|  | 2 | 2.44 | 0.56-10.6 | 0.237 |
|  | 3 | 4.24 | 0.81-22.3 | 0.088 |
| Classification | NET | 1 |  |  |
|  | NEC | 2.09 | 0.67-6.51 | 0.202 |
|  | Non-NEN | 0.93 | 0.40-2.19 | 0.877 |

HR: Hazard ratio; CI: Confidential interval
